# The indirect impact of control measures in COVID-19 pandemic on the incidence of other infectious diseases in China

**DOI:** 10.1016/j.puhip.2022.100278

**Published:** 2022-06-13

**Authors:** Shuangshuang Song, Ping Wang, Jian Li, Xiuzhen Nie, Liyan Liu, Shihua Liu, Xiuzhi Yin, Aiwei Lin

**Affiliations:** Infectious Diseases Department, Jinan Children's Hospital: Children's Hospital Affiliated to Shandong University, No. 23976 Jingshi Road, Jinan, 250022, Jinan, China

**Keywords:** COVID-19, Prevention control measures, Impact on other infectious diseases

## Abstract

**Objectives:**

During COVID-19 pandemic in 2020, China, some public health measures of forced lockdown, closure of school and public meeting places, staying at home, transportation stop, masks wearing, hands washing, environmental disinfection were taken on to control epidemic transmission, these measures have made indirect affect on the other infectious diseases incidence.

**Study design:**

During COVID-19 pandemic in 2020, we retrospectively analyzed and compared reported cases of other infectious diseases,in order to found what impact of measures in controlling COVID-19 pandemic on the other infectious diseases in China.

**Methods:**

We retrospectively analyzed and compared reported cases of measles, pertussis, scarlet fever, seasonal influenza, mumps, HFMD each month in 2018, 2019 and 2020 from the National Health Commission, PRC.

**Results:**

Cases of measles, pertussis, scarlet fever, seasonal influenza, mumps and HFMD in January 2020 were not declined, or even increased compare to 2018 and 2019, but from February to December 2020, began to drop significantly compare with the cases of 2018 and 2019. However, seasonal influenza cases in 2020 were more than in 2018.

**Conclusion:**

It shown that how important scientific measures are taken to cut off COVID-19 pandemic transmission, However, these taken measures have led to indirect impact on the diffusion of other infectious diseases, led to measles, pertussis, scarlet fever, seasonal influenza, mumps, HFMD declined.

## 1 Background

In December 2019, the outbreak of the Coronavirus disease 2019 (COVID-19) was firstly reported in Wuhan, China [1,2], and the disease has spread rapidly throughout China and the rest of world. Although the main transmission route for COVID-19 is droplets, during aerosol generating procedures airborne transmission may occur [3]. Simultaneously, the positive effects brought by the COVID-19 pandemic were the high commitment of citizens' practice of hand washing, mask wearing, and personal hygiene and raised awareness of other preventive measures, which have brought windows of opportunity not only to decrease pediatric admissions due to respiratory diseases [4], but to incorporate public health science into public policies. The measures adopted to contain the pandemic are having a significant economic impact, but they may have indirect effects on the diffusion of other infectious diseases, particularly epidemic diseases of childhood [5]. In China, many paediatric infectious disease physicians found that a sharp decline in paediatric admissions for respiratory illnesses and infectious diseases, such as measles, pertussis, scarlet fever, seasonal influenza, mumps, hand-foot and mouth disease (HFMD). Measles is a highly contagious, acute febrile illness that results from infection with measles virus, Measles transmission occurs person-to-person via large respiratory droplets and via airborne transmission of aerosolized droplet nuclei in closed areas (e.g., an office examination room) for up to 2 h after a person with measles occupied the area. Pertussis is a highly contagious acute respiratory illness classically known as “whooping cough” because of its characteristic cough. The majority of cases are caused by Bordetella pertussis, with some caused by B. parapertussis. Scarlet fever is an infectious disease caused by toxin-producing strains of Streptococcus pyogenes, transmission by the respiratory mode, bacteria commonly found on the skin or in the throat, where they can live without causing problems. However, under some circumstances, they can also cause diseases such as scarlet fever. seasonal influenza is an acute respiratory disease in mammals and domestic poultry. Spread of seasonal influenza virus is through coughing, sneezing, talking (within 6 ft), or coming into contact with contaminated surfaces, also including be transmitted aerosol form (short range airborne infection). It's virus is made up of 8 distinct RNA segments. These segments each code for a different protein that is essential to the structure and function of the virus. There are 3 genera of seasonal influenza viruses: A, B, and C,Antigenic drift and antigenic shift are continuous processes that result in seasonal influenza viruses existing as a quasispecies [6]. Mumps is a common childhood infection caused by the mumps virus. The hallmark of infection is swelling of the parotid gland. Transmission of the virus is by direct contact, droplet spread, or contaminated fomites. HFMD is caused by human enteroviruses, it is transmitted by fecal-oral, oral-oral, and respiratory droplet contact.

Above infectious diseases are mostly transmitted through coughing, sneezing, talking, or coming into contact with contaminated surfaces, also including aerosol form (short range airborne infection). In order to presents the epidemiological transition of measles, pertussis, scarlet fever, seasonal influenza, mumps and HFMD during the pandemic, we collected the cases reported in 2018–2020 through China to explore measures for controlling infectious diseases incidence during and after COVID-19 pandemic.

## 2 Methods

We obtained nationwide reported infectious diseases including measles, pertussis, scarlet fever, seasonal influenza, mumps, HFMD each month from 2018 to 2020 from the Official Website of Bureau for Disease Control and Prevention in National Health Commission of People's Republic of China [7]. Retrospectively analyzed and compared all confirmed infectious diseases cases. Compared reported cases of 2018 and 2019 to 2020 using the chi-square test. 2020 cases lower rate than that of 2018 and 2019 was described using percentages. Significance was set at a P-value of <0.05. All analyses were performed using Statistical Package for Social Science (IBM SPSS software, USA) version 16.0.

## 3 Results

From February to December in 2020, the reported cases of measles, pertussis, scarlet fever, mumps cases began to drop significantly compare with the same period in 2018 and 2019 (Supplementary Fig. 1). It can be seen that total cases of measles, pertussis, scarlet fever, mumps, HFMD in 2020 marked lower than that in 2018 and 2019, but seasonal influenza reported cases in 2020 were more than in 2018 (Table 1). Measles in 2020 was 72.5% and 65.5% lower than in 2018 and 2019, respectively. Pertussis was 77.8% and 83.7% lower than in 2018 and 2019. Scarlet fever was 78.5% and 79.3% lower than in 2018 and 2019. seasonal influenza was −59.7% and 65.0% lower than in 2018 and 2019. Mumps was 51.1% and 57.8% lower than in 2018 and 2019. HFMD was 67.6% and 60.4% lower than in 2018 and 2019.

**Table 1:** Reported cases of Measles, pertussis, scarlet fever, influenza, mumps and HFMD in three years in China.

Disease/Year	2018	2019	2020	2018-2020/2018	2019-2020/2019	abP
**Measles**	4483	3573	1234	3249 (72.5%)	2339 (65.5%)	<0.001
**Pertussis**	22466	30727	4994	17472 (77.8%)	25733 (83.7%)	<0.001
**Scarlet fever**	79845	83028	17206	62639 (78.5%)	65822 (79.3%)	<0.001
**Influenza**	768291	3507306	1226804	−458513 (−59.7%)	2280502 (65.0%)	<0.001
**Mumps**	261493	303105	127807	133686 (51.1%)	175298 (57.8%)	<0.001
**HFMD**	2375938	1944036	769448	1606490 (67.6%)	1174588 (60.4%)	<0.001

a Comparison of infectious diseases cases between 2018 and 2020.

b Comparison of infectious diseases cases between 2019 and 2020.

## 4 Discussion

During the pandemic of COVID-19 throughout China, the infectious diseases of measles, pertussis, scarlet fever, seasonal influenza, mumps and HFMD sharply declined. In order to presents the epidemiological transition, we collected the cases reported in 2018–2020 through China. COVID‐19 caused by the virus severe acute respiratory syndrome-coronavirus-2 (SARS-CoV-2), it is a novel disease declared a pandemic by the Word Health Organization on March 11, 2020. The COVID-19 epidemic was limited to Wuhan or/and Hubei in the early and middle of January 2020, and there were no COVID-19 cases in other places in China. Therefore, no strict prevention and control measures were taken in other places except Wuhan or/and Hubei, so that other infectious diseases were still frequent. In late January, with the arrival of the Spring Festival, many people in Wuhan or/and Hubei returned to their hometown, COVID-19 began to develop in many places across the country, so some public health measures to prevention and control pandemic became stricter: forced lockdown, closure of school and public meeting places, staying at home, transportation stop, masks wearing, hands washing, environmental disinfection, and so on. While the impact of these measures on the spread of other infectious diseases is also becoming apparent. In this context, the opportunities for contagion: measles, pertussis, scarlet fever, seasonal influenza, mumps and HFMD, have significantly reduced, with favorable repercussions on the spread of common epidemic diseases, particularly among children, which also happened in other country [8]. Furthermore, an intensive use of masks in general population could have reduced the inhalation of airborne respiratory droplets, which are the main vehicle for spreading some of these diseases, because these diseases are mainly transmitted through the respiratory tract. Another explanation is that the cases registered in Infectious Diseases Department could underestimate the total cases. COVID‐19 epidemic indeed led to a decreasing flow to the Infectious Diseases Department, due to a common fear of being infected during Infectious Diseases Department in-stay.

During COVID-19 pandemic, the reported cases of seasonal influenza in 2020 were significantly lower than in 2019, however, higher than in 2018. it may be due to 2018 was a small flu year. Cases of measles, pertussis, scarlet fever, mumps and HFMD had significantly decreased in 2020 compare to 2018 and 2019. It suggesting that the transmission dynamics of these diseases were occluded by taken prevention measures. While vaccination might also have partly contributed to this decrease. But to such obviously declined infectious diseases, we argue that there were several potential reasons for this achievement that brought by the COVID-19 pandemic. First, enhanced hygiene measures such as cough etiquette and regular mask wearing decreased the transmission of these infectious diseases [9,10]. Second, the increasing implementation of work from home (i.e., teleworking), it could be assumed that decreasing human mobility and social contacts in workplaces, which became more common in response to the COVID-19 pandemic [11], might have positively contributed to interrupting the transmission dynamics of these diseases. Finally, reduce crowd gathering also contributed to the decrease. Therefore, in addition to vaccination, it is very important to actively control the transmission of infectious diseases, such as measles, pertussis, mumps, seasonal influenza and HFMD vaccination, and so on. However, the vaccination can not completely block and prevent the occurrence of infectious diseases.

The purpose of this report is to illustrate the prevention measures taken in China impact on the occurrence of other infectious diseases after outbreak of COVID-19 pandemic. Because China is a large population and developing country, and the prevention measures taken in China are more rigorous and prolonged, and the policy of “dynamic zero” is implemented when COVID-19 occurred in one area, which different from other countries and regions. From the indirect effects of prevention measures of COVID-19 on the infectious diseases, that shown how important to control the source of infection, cut off transmission routes, and protect vulnerable populations in prevention infectious diseases, in particular, scientific policy measures are taken to cut off transmission routes. But there are some limitations in this study, we only discussed the effect of public health measures on infectious diseases, not drug treatment, nor the annual prevalence of infectious diseases.

## Author statements

The authors thank the National Health Commission, PRC.

## Ethics approval

This report approved by the Institutional Ethics Board of Jinan Children's Hospital: Children's Hospital Affiliated to Shandong University.

## Funding

None declared.

## Competing interests

None declared.

## Declaration of competing interest

The authors have declared that no conflicting interests exist.

## Appendix A Supplementary data

figs1 figs1
