# Supplementary figures and images for: The indirect impact of control measures in COVID-19 pandemic on the incidence of other infectious diseases in China
Source: Public Health Pract (Oxf). 2022 Jun 13;4:100278. doi: 10.1016/j.puhip.2022.100278 (PMC9190177; doi:10.1016/j.puhip.2022.100278)

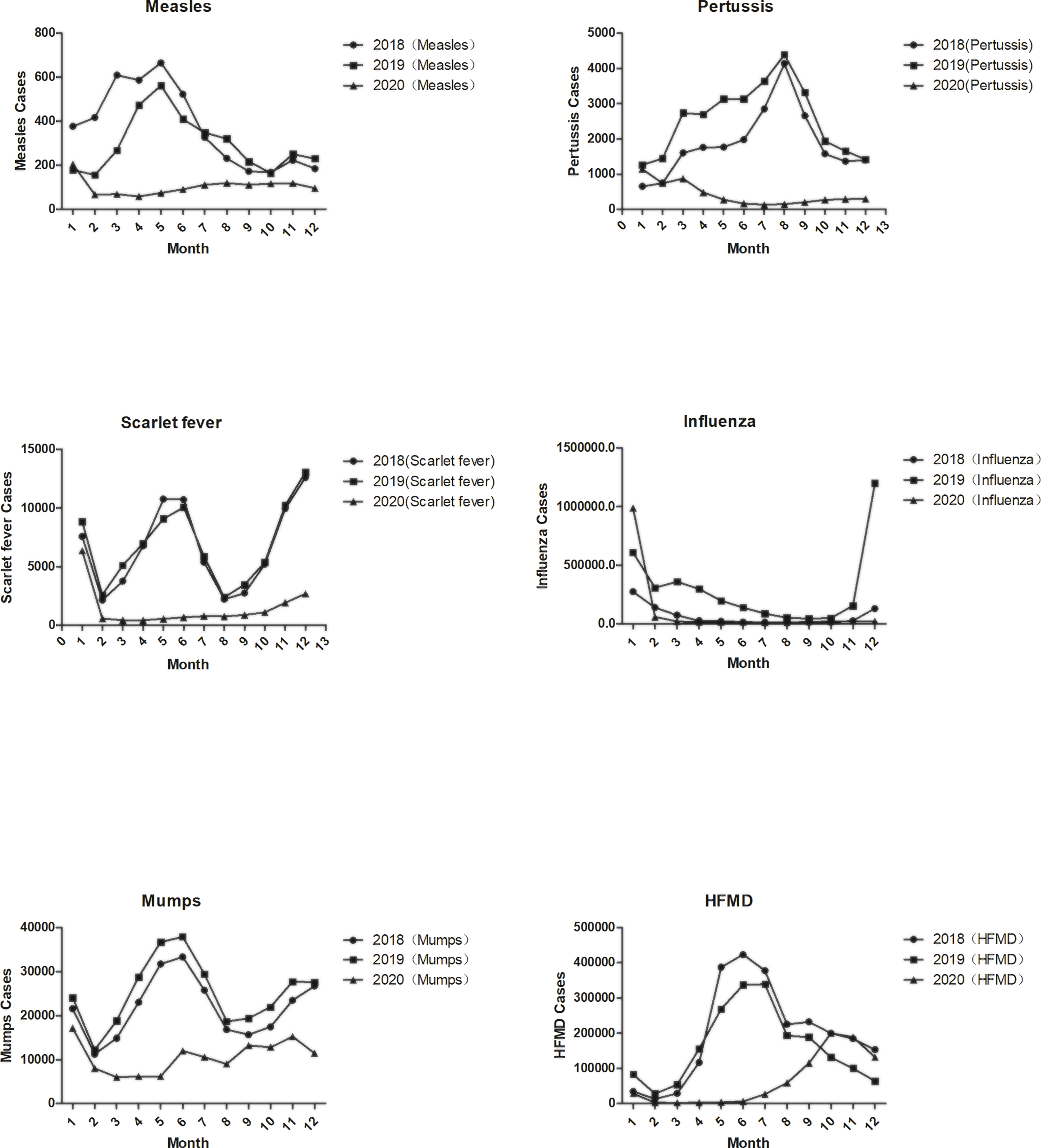

Supplement: figs1 [file mmcfigs1.jpg]
